# Gender in the Afterlife: An Exploration of Dynamic Gender Stereotypes in the Epitaphs of the Merry Cemetery of Săpânţa

**DOI:** 10.3389/fpsyg.2018.01436

**Published:** 2018-08-08

**Authors:** Petru L. Curşeu, Ioan Pop-Curşeu

**Affiliations:** ^1^Department of Psychology, Babeş-Bolyai University, Cluj-Napoca, Romania; ^2^Department of Organisation, Open University of the Netherlands, Heerlen, Netherlands; ^3^Department of Cinematography and Media, Babeş-Bolyai University, Cluj-Napoca, Romania

**Keywords:** gender stereotypes, femininity, masculinity, social roles, Romania

## Abstract

Gender stereotypes are shaped by the roles men and women fulfill in society. Our study uses a cultural artifact analysis and explores the way in which remunerated jobs, development across lifespan and historical time frames influence the content of gender stereotypes. We coded the feminine and masculine attributes in a selection of epitaphs written on the painted crosses of the Merry Cemetery of Săpânţa (Romania) between 1935 and 2010. This novel historical approach allowed us to explore the dynamic nature of gender stereotypes and the extent to which changes in the social context or the social roles have transformed the content of gender stereotypes. We show that during years of social unrest associated with the World War II and the early communist years the masculine attributes are dominant while during the last decades of the communist regime and the post-communist period the feminine attributes become more prevalent. Moreover, people having paid jobs are described as being more masculine than the homemakers. Finally, our results show an increase of masculinity during the lifespan for both male and female as well as an increase of androgyny with age for women and a slight decrease with age for men.

## Introduction

Gender stereotypes reflect general expectations about typical men and typical women (Ellemers, 2018) and a central claim in social role theory is that stereotypes change in time and when social context changes (Eagly et al., 2000). More specifically, the changes in the content of stereotypes follow the changes of typical activities associated with a particular social role (Diekman and Eagly, 2000). For example, as women participation in the workforce substantially increased in the last decades (also in jobs that used to be male dominated) the content of socially shared gender stereotypes changed as well and modern women tend to be described as more “masculine” than women of the past (Echabe and Castro, 1999; Diekman and Eagly, 2000; Wilde and Diekman, 2005; Donnelly and Twenge, 2017; Bosak et al., 2018).

A typical procedure used to explore dynamic stereotypes is to ask participants to describe people as they were typical in the 50s’, 70s’ or current time. Although informative on the difference in gender related attributes ascribed to women and men across various time frames, this method is subject to recollection biases and it does not allow an accurate evaluation of the real stereotypic content associated with men and women. Moreover, empirical research, rarely captures simultaneously the effect of historical changes, gender and age on the dynamics stereotypes content. Historical effects can be attributed to how specific social roles change in different historical periods, while age effects reflect the change that can be attributed to specific role changes across the lifespan of individuals (i.e., role accumulation). In other words, change in the content of gender related stereotypes can be attributed to the fact that in time, the generic content of the gender social roles changes (e.g., due to the increase labor force participation of women during the last decades, Donnelly and Twenge, 2017) or to the specific difference in the social roles of individual members in a community across their lifespan (e.g., the differences between the gender stereotypes used to describe adolescents versus older adults Strough et al., 2007). These cross-temporal and cross-demographic comparisons are also restricted due to limited data availability (especially earlier than 1960s).

**FIGURE 1:**
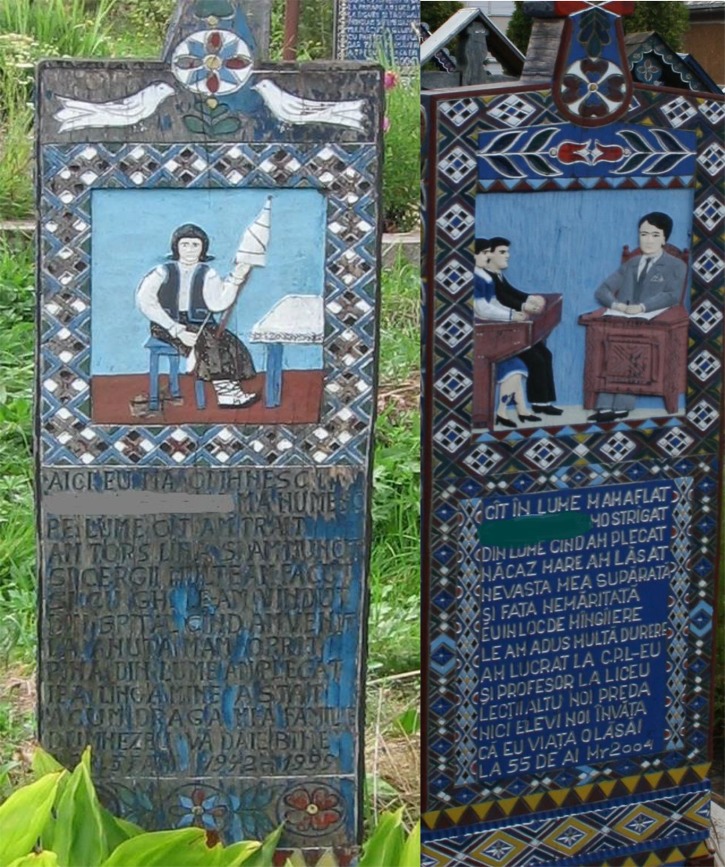
Examples of occupations depicted on the painted crosses of the Merry Cemetery of Săpânţa. Translation of the epitaph on the left: *Here I lie to take my rest/And SI is my name/As long as I lived/I worked by hand spinning the wool/And I weaved many rugs/Sold them with GH/When from hospital I returned/Anuta took good care of me/Until I left this world/And now my dear family/I wish God will bless you all*; Translation of the epitaph on the right: *As long as I lived/People called me OP/When I departed from this world/Misfortune I left behind/An upset wife/And an unmarried daughter/Instead of comfort/I brought them only pain/I worked at CPL and I was also a high school teacher/ But I will teach no longer/Nor will I guide pupils/As I died at 55.*

We suggest that the use of a historical approach that rates the content of gender stereotypes in cultural artifacts can be a useful approach to investigate the content of dynamic gender stereotypes. Such cultural artifacts are rich repositories of valuable social knowledge that can be used to make inferences on the dynamics of social cognition (Curşeu and Pop-Curşeu, 2011). Moreover, previous research using non-invasive, historical approaches shows that obituaries as short biographical descriptions can be used to capture the way in which the content of stereotypes changes in time (Kirchler, 1992; Rodler et al., 2001; Curşeu and Boroş, 2011). We therefore set out to explore using this novel historical approach gender stereotypes recorded in a cultural artifact claimed to be a social knowledge repository in a small Romanian community (Curşeu and Pop-Curşeu, 2011). The epitaphs written on the blue-painted crosses of the Merry Cemetery of Săpânţa summarize the identities of deceased community members dating back to the 1930s. Examples of painted crosses with the written epitaphs are presented in **Figure 1**.

In this paper we answer the call for more ecological investigations of how stereotypes actually change over time (Diekman and Eagly, 2008; Haines et al., 2016; Ellemers, 2018) and we aim to investigate the way in which age, the social roles and historical periods (illustrated by the year of death), influence the content of gender stereotypes used in the epitaphs to describe the deceased community members. This ecological and historical perspective allows us to capture time-bounded snapshots of personal biographies and isolate the historical period effects from age or social roles-related effects on stereotype change.

### Dynamic Gender Stereotypes

Initial research on the content of gender stereotypes claimed that the beliefs related to gender differences are stable across life stages and rather stable in time (Hilton and von Hippel, 1996). Recent approaches theorized gender stereotypes as dynamic constructs and show that some of the gender specific attributes associated with men and women might change in time due to the inherent changes in societal dynamics and social roles (Diekman and Eagly, 2000). As Diekman and Eagly (2000) claim, these results are aligned with the conception of individuals as implicit social role theorists that observe transitions in gender related expectations that are closely related to social changes.

People act as implicit role theorists and make inferences about the typical attributes of men and women depending on the dominant social roles each social category tends to fulfill. In the past, women were mostly observed in domestic, household-related roles and as such were attributed more communal traits, while men were observed more in “breadwinner” roles, and were ascribed more agentic traits (Bosak et al., 2018). As the social and historical contexts are changing, these changes are expected to re-shape the roles men and women take. Using a typical design for studying dynamic gender stereotypes Banchefsky and Park (2016) show that mothers and fathers of the future tend to be seen as more similar than they were seen in the past. These changes are explained by the fact that men and women are expected to assume more similar social roles in the future. In other words a key tenet of dynamic gender stereotypes is that the change in the social context is associated with changes in the descriptive norms and attributes ascribed to men and women (Diekman and Eagly, 2000; Bosak et al., 2018). At least three perspectives co-exist with respect to the change in the social context: (1) a historically driven change, in the content of gender stereotypes as a consequence of changes in the differential access to specific social roles that men and women have across different time periods (historical effect), (2) a change associated with the accumulation of various roles during the lifespan (age effect) and (3) a change in the adoption of specific social roles by various individuals.

#### Gender Influences

Gender schema theory (Bem, 1977) posits that social categorization processes trigger gender schematic information processing, leading to specific stereotypical attributes being associated with men and women. Individuals belonging to the same social category tend to share more similar attributes than they do with individuals that belong to different social categories. In line with the depersonalization principle in social categorization (Hornsey, 2008), while organizing the information about their social world, people use generic (impersonal) gender-typed schemas to attribute gender specific characteristics to individuals they categorize as men or women. According to these social categorization claims, the gender schema theory (Bem, 1977) states that men should (in principle) be described more in terms of masculine traits, while women should (in principle) be described using feminine attributes. In Romanian rural communities men are seen as more inclined to take public roles, while women are seen as being more active in the family-related roles (Curşeu and Boroş, 2011). The specific context in which we could explore the content of the cultural artifacts is a rural community in which the gender roles still follow a traditional distinction with women more present in domestic/homemaker roles, while men are more present in public/breadwinner roles. In line with these theoretical arguments, we expect that in the epitaphs of the Merry Cemetery of Săpânţa (MCS), the incidence of feminine attributes will be higher for women than for men, while the incidence of masculine attributes will be higher for men than for women (*Hypothesis 1*).

#### Influences of Historical Periods

Social events that unfold during history bring forth changes in the way individuals experience life (Stewart and Healy, 1989) and ultimately impact on their individual attributes, personality and identity (Stewart and Ostrove, 1998). Moreover, Sewell (1996) argues that major historical events trigger changes in social structures and institutions. As individuals live their lives in intricate social structures (Curşeu and De Jong, 2017) and (must) comply with various institutional norms, the change of these social structures and institutions triggers changes in personal identity as well. We build on these theoretical concepts that tie personal development to historical changes and argue that gender attributes, as prototypical representations of social roles attributed to men and women, change as a function of major historical events. In particular, critical social events such as wars tend to favor masculine attributes, therefore the use of masculine traits is expected to dominate during times of social unrest such as wars and social conflicts, while feminine attributes are expected to be associated with social stability. Starting with the 1930s when the practice of writing epitaphs on painted crosses at Săpânţa was initiated by Ioan Stan Pãtraş (Curşeu and Pop-Curşeu, 2011) three major historical events in the sense described by Sewell (1996) can be identified. The first one is the period surrounding the World War II, a time of social unrest and conflict that affected virtually the whole Romanian territory. The second major historical change is the instauration in the late 1940s and domination until 1989 of the communist regime. Previous research explored the implications of communism ideology for gender discrimination and sexism (Curşeu and Boroş, 2011; Zawisza et al., 2012, 2015). The communist era in Romania was initially marked by social unrest and resistance against the new regime (especially in rural areas in which the nationalization of private property generated great opposition) and then after 1967, during the Ceauşescu regime, marked by a pervasive propaganda focused on social equality and nationalism. This period was also influenced by the propagandistic image of the “heroic mother” and the marked public presence of Ceauşescu’s wife as idealized image of the “mother of the nation.” Finally, after 1989, the post-communist era reflects a transition to a market economy and adoption of capitalist values and social practices. In line with these historical events, we expect that masculine and instrumental attributes will dominate in the period surrounding WWII and the early turbulent years of the communist regime, while during Ceauşescu regime under the intense propaganda that favored feminine attributes and equality, we expect feminine attributes will become more prevalent in the epitaphs of the MCS. In other words, we expect a positive association between femininity (and not masculinity) and year of death (*Hypothesis 2*).

#### Social Roles Influences

Social role theory posits that people describe themselves and are described by others in line with the social roles they occupy (Eagly, 2013). Previous research has shown that the increase in participation of women in the paid labor force fosters the ascription of masculine attributes to women (Lopez-Zafra and Garcia-Retamero, 2012). Moreover, in general employees tend to be characterized by masculine rather than feminine attributes when compared with homemakers (Eagly and Steffen, 1984). This effect was gender insensitive, in that both men and women with a paid profession were described using masculine rather than feminine traits (March et al., 2016). In rural communities, employed people have to often combine their professional roles with household activities and additional social roles, therefore we also expect a role accumulation effect for people with paid professions. In line with these arguments and also in line with the claims of dynamic stereotypes perspective (Diekman and Eagly, 2000) we argue that people engaged in paid work (employed) will be described as being more masculine and androgynous than homemakers (*Hypothesis 3*).

#### Age Influences

Role accumulation across the lifespan was conceptualized as a source of gratification as various roles assumed simultaneously by individuals bring multiple role privileges, offer multiple resources for building and maintaining social status and contribute to the personality and ego enrichment (Sieber, 1974). In line with these role accumulation arguments (in particular the personality and ego enrichment), as individuals may take multiple roles simultaneously, another hypothesis derived from the social roles theory is that the number of attributes used to describe a particular person proportionally increases with the number of roles that a person takes. In a developmental perspective, as they grow older, individuals accumulate various roles and as roles and life experiences accumulate during ones lifespan, it is also more likely that androgyny increases with age. In line with these arguments we therefore expect that the incidence of both men specific and women specific attributes in the epitaphs of the MCS will increase with the age of the deceased person (*Hypothesis 4*).

Literature to date explored the interplay between societal changes and the changes in the content of gender stereotypes. Societal changes bring forth changes in typical gender roles as well as changes in social status for men and women. Diekman et al. (2005) compared the content of dynamic stereotypes in US, Chile, and Brazil. Their results show a steady increase in women’s perceived masculinity in all three cultures (linked to women’s increased presence in the labor market and public roles), while for men this increase was observed in Chile and Brazil and was associate to the societal changes specific to these countries (Diekman et al., 2005). In another study on the dynamics of power in relation to gender Diekman et al. (2004) show that the perceived power increases steadily for women, while for men remains relatively stable. For a non-western cultural context, Bosak et al. (2018) show gender differences in the historical period effects such that in time, Ghanian women tend to be perceived as being more masculine, while men are perceived as gaining in femininity.

In a cross-temporal meta-analytic investigation of Bem Sex Roles Inventory (BSRI) scores Twenge (1997) reports gender specific and significant cohort effects for masculinity (women’s masculinity increases between 1973 and 1994 with the year in which the data was collected). A more recent meta-analysis on the cross-temporal dynamics of the BSRI scores shows a decline in women’s femininity between 1993 and 2012, while their masculinity did not increase in this time interval (Donnelly and Twenge, 2017). To summarize, the results to date suggest that gender might qualify the effect of age, profession and historical period as they were hypothesized earlier. However, as the moderating role of gender is closely related to the specific societal, historical and cultural context in which it is investigated, we set out to explore this moderating role, without formulating specific hypotheses.

## Ethics Statement

This study is based on artifact analysis and it involved the analysis of public data. Although the data reflects individual biographies, none of the participants were alive at the time of the study. In the data sets on which analyses were carried out participants were anonymized and because no additional harm was inflicted on the participants we did not seek an IRB approval for this study.

## Materials and Methods

### Gender Stereotypes Evaluation

To evaluate gender-role stereotypes we have adapted items from the Bem Sex Role Inventory (BSRI, Bem, 1974) because this measure was extensively used in various cultural contexts (Holt and Ellis, 1998). Although BSRI was previously used to evaluate both gender identity and gender stereotypes, given that the content of the items largely overlaps with the content of gender stereotypes (Spence and Buckner, 2000) we used the BSRI attributes as indicators of gender stereotypes present in the epitaphs. Moreover, because in our study we have analyzed the content of a cultural artifact, consisting of short biographical sketches made by third parties and we do not rely on self-reports, the results based on the BSRI reflect stereotypes contents and not gender identity. We have used research on gender stereotypes carried out in Romania (Curşeu and Boroş, 2011; Ivan, 2012; Stan and Secui, 2012) to find gender specific attributes for men and women identified in this particular cultural context. We started by exploring previous analyses using the BSRI on Romanian samples (Stan and Secui, 2012; Ivan, 2012) and selected the most representative items (based on their factor loadings when available) for the male/female role description. As previous research points out that in traditional Romanian communities (Curşeu and Boroş, 2011; Curşeu and Pop-Curşeu, 2011; Stanciu et al., 2017), women are perceived as being strongly involved in the family/private domain, while men as being strongly involved in the public sphere, we also included two attributes “devoted to family” (typical female) and “devoted to community” (typical male) to the items selected from the BSRI. Some of the items used in the short form of BSRI were adapted for example, “leadership ability” does not differentiate among men and women in the Romanian context as incidence of women leaders was rather high during the communist regime (Curşeu and Boroş, 2011) and both men and women specific attributes are associated with effective leadership styles (Ivan, 2012). We therefore used “takes the initiative/leads” as a male typical attribute. Moreover, in traditional Romanian communities, women are perceived as being more religious than men are (Curşeu and Pop-Curşeu, 2011) therefore, “religious” was added as a typical feminine attribute. The final selection of items included 15 male and 15 female typical attributes. The attributes and their respective factor loading in the first two dominant factors (factor 1 – typical male attributes and factor 2 – typical female attributes) are presented in **Table 1**.

**Table 1:** Factor loading for the first two dominant Bartlett scores.

BSRI item	First dominant factor masculinity	Second dominant factor femininity
1. Devoted to community (M1)	0.285	0.033
2. Independent (M2)	**0.764**	-0.068
3. Aggressive (M3)	0.062	-0.160
4. Physically resistant (M4)	**0.356**	-0.159
5. Makes decisions easily (M5)	**0.735**	0.134
6. Self-confident (M6)	**0.823**	-0.100
7. Never gives up easily (M7)	**0.645**	0.138
8. Skillful (M8)	**0.649**	-0.044
9. Industrious (M9)	**0.656**	-0.014
10. Takes the initiative/leads (M10)	**0.822**	0.070
11. Analytical/Logical (M11)	**0.334**	0.078
12. Defends own beliefs (M12)	**0.643**	-0.129
13. Self-reliant (M13)	**0.854**	-0.148
14. Determined (M14)	**0.791**	-0.035
15. Stand up well under pressure (M15)	**0.499**	0.227
16. Devoted to family (F1)	-0.133	**0.596**
17. Interdependent/relational (F2)	-0.436	**0.426**
18. Sensible (F3)	0.211	**0.333**
19. Fragile (F4)	-0.342	0.224
20. Pays more attention to details (F5)	0.370	0.285
21. Gentle (F6)	-0.193	0.212
22. Accepting (F7)	0.028	0.109
23. Nurturing (F8)	0.280	**0.688**
24. Compassionate (F9)	0.097	**0.739**
25. Mother/loves children (F10)	0.233	**0.733**
26. Affectionate (F11)	-0.104	**0.790**
27. Eager to soothe hurt feelings (F12)	-0.027	**0.619**
28. Sensitive to the needs of others (F13)	0.217	**0.658**
29. Warm in relations to others (F14)	-0.115	**0.680**
30. Religious (F15)	0.079	0.239


### Epitaphs Sampling and Coding

We have used a stratified selection procedure (gender, age, and time frame) to compile the list of epitaphs to be coded and further analyzed. We selected 197 epitaphs (101 women) of community members deceased between 1935 and 2010, with ages up to 96 years old. A problematic category was the pre-adolescence age group that was underrepresented in the final sample of epitaphs, yet the sample also included deceased newborn infants. We however, deemed relevant to include adolescence and pre-adolescence as well, in order to have a more complete picture of the lifespan variation of gender stereotypes. An independent coder, unaware of the specific hypotheses of the study coded each of the epitaphs using the BSRI attributes. We have used an integrative coding procedure, such that synonyms for the BSRI attributes, or implied attributes from selected biographical facts, were also considered in the coding scheme. Each attribute from the list that was deemed to be present in the epitaph was coded with one and the ones that were not present in the epitaph were coded with zero.

## Results

### Preliminary Analyses

As the meta-analytic investigation of Choi and Fuqua (2003) showed that the factorial structure of the BSRI might not be stable across contexts (cultural and time related) we started by analyzing the factorial structure of the BSRI for our study. We have used PAF (Principal Axis Factoring from the covariance matrix without any rotation) that revealed two dominant factors that cover together more than 47% of the variance in the scores. The first dominant factor had an eigenvalue of 1.62 and covers 27.8% of variance in scores, while the second dominant factor has an eigenvalue of 1.12 and covers 19.28% of variances in scores. We have used the results of the factor analysis (on the item covariance matrix) to compute two dominant factor scores and in order to obtain scores as close as possible to the “true factor scores” we have used the Bartlett’s approach (DiStefano et al., 2009). Although in the Bartlett approach the extracted factors may still be correlated, the scores are indicative of the true factor evaluated by the items included in the scale (DiStefano et al., 2009). The two dominant regression factor scores obtained in our analyses were uncorrelated and the item loadings in the two dominant factors are presented in **Table 1**. Most of the items load, as expected on their respective factor score, the masculine items load in the first factor, while most of the feminine items load on the second one (the items with factor loading higher than 0.33 are presented in bold). The two resulting dominant factor scores were saved as variables and used as indicators of masculinity and femininity in subsequent analyses as they are accurate (underlying) factor estimators to which each of the BSRI items contribute with its individual factor load.

In line with previous research we have used three main indicators derived from the BSRI.

*Masculinity* was evaluated using the first dominant factor score that resulted from the factor analysis while *femininity* was evaluated using the score of the second dominant factor. As Bartlett scores are likely to be indicative of the true factor scores (DiStefano et al., 2009), in what follows we will report the results of the analyses using the Bartlett dominant factors scores derived from covariance matrix in the factor analyses using PFA.

However, because the regression procedure for computing the dominant factor scores maximized the validity of the estimates (DiStefano et al., 2009) we also used this procedure for computing the factor scores, in order to cross check the results.

*Androgyny* was computed using the procedure described by Donnelly and Twenge (2017), namely we subtracted the value of the absolute difference between masculine and feminine attributes that loaded in their respective factor from the total number of attributes [Androgyny = (M + F)-|M-F|]. To compute the androgyny score, we kept the scores for the items presented in bold in **Table 1** and a high score for androgyny reflects the co-existence of both masculine and feminine traits.

In order to make sure that the coding of the epitaphs was accurate, we asked a second coder to evaluate forty epitaphs coded by the main coder and we have computed the intra-class correlation coefficient in order to correct for agreement by chance (MacLennan, 1993). For the masculinity attributes, the intra-class correlation coefficient was 0.87 and the Chi-square was 5.96 (ns), while for the femininity scores the intra-class correlation coefficient was 0.84 and the Chi-square was 3.59 (ns) showing evidence of substantial agreement between the two coders. Based on this substantial inter-coder reliability, we have used for further analyses the coding of a single independent coder. The resulting codes were further checked for consistency and for the masculinity scale, Cronbach’s alpha is 0.90 while for the femininity scale, Cronbach’s alpha is 0.83. We can therefore conclude that the coding procedure resulted in reliable estimates of masculinity and femininity.

### OLS Regression Analyses

Means, standard deviations and correlations for the variables included in the study are presented in **Table 2**.

**Table 2:** Means, standard deviations, and correlations for study variables.

	Mean	*SD*	1	2	3	4	5	6
(1) Gender	0.55	0.49						
(2) Age	43.46	26.97	0.25^**^					
(3) Year of death	1975.48	18.36	0.02	0.18^*^				
(4) Paid profession	0.25	0.43	-0.11	0.17^*^	0.24^**^			
(5) Masculinity	0.00	1.02	-0.09	0.50^**^	0.16^*^	0.49^**^		
(6) Femininity	0.00	1.04	0.41^**^	0.16^*^	0.26^**^	0.00	-0.00	
(7) Androgyny	5.12	5.78	0.10	0.32^**^	0.24^**^	0.33^**^	0.66^**^	0.53^**^


*^∗^p < 0.05; ^∗∗^p < 0.01. ^∗∗∗^p < 0.001. Correlation coefficients are presented in the table, Gender is coded as a dummy variable with 1 = women and 0 = men, Paid profession is coded as a dummy variable with 1 = has a paid profession and 0 = does not have a paid profession.*

In order to test the hypotheses we have used OLS regression analyses with femininity, masculinity and androgyny as dependent variables. As predictors, we have entered in the first step (Model 1 in **Table 3**) gender, paid profession, year of death. Based on a suggestion received during the review process, we accounted in the second step (Model 2 in **Table 3**) for the plausible non-linear effect of age as well as for the interaction between gender and age, year of death and paid profession. It is likely that role accumulation reaches a maximum in the late adult life and as such androgyny reaches a plateau around the age of 60. Moreover, the effect of historical periods (year of death), age and profession might show a gender dependency as mentioned in the theoretical arguments. In order to account for these potential effects, we have entered the squared term for age and the interaction term between gender and all the other between subjects variables in the second step of the analyses. Age and year of death were grand mean centered before computing the cross-product terms and the squared term for age (as gender and paid profession are dummy variables, we did not center them before computing the cross-product terms). The results of these analyses are presented in **Table 3**. For illustrative purposes we also depict in several figures the slopes for masculinity, femininity and androgyny.

**Table 3:** Results of the OLS regression analyses.

Predictors	Masculinity		Femininity		Androgyny	
			
	Model 1	Model 2	Model 1	Model 2	Model 1	Model 2
Gender	-0.17^**^	-0.28^***^	0.39^***^	0.45^***^	0.07	0.02
Paid profession	0.39^***^	0.20^*^	-0.02	0.05	0.27^***^	0.16†
Year of death	-0.02	0.06	0.26^***^	0.24^**^	0.13†	0.21^*^
Age	0.48^***^	0.51^***^	-0.02	-0.10	0.24^**^	0.07
Gender X Year of death		-0.09		0.02		-0.10
Gender X Age		-0.04		0.18†		0.24^*^
Gender X Paid profession		0.27^**^		-0.16†		0.13
Squared age		-0.09†		-0.12†		-0.12†
R^2^	0.44	0.48	0.23	0.26	0.20	0.25
F Change	38.13^***^	3.72^**^	14.34^***^	2.01†	12.09^***^	2.85^*^


*^†^p < 0.10. ^∗^p < 0.05; ^∗∗^p < 0.01. ^∗∗∗^p < 0.001. Standardized regression coefficients are presented in the table; Gender is coded as a dummy variable, 0 = men, 1 = women.*

Gender had a negative and significant effect on masculinity β = -0.28 (*p* < 0.001) and a positive and significant effect on femininity β = 0.45 (*p* < 0.001) showing that, in line with Hypothesis 1, women were characterized by more feminine than masculine attributes and the opposite is observed for men. Therefore, we can conclude that Hypothesis 1 was supported. Gender differences were however not significant for androgyny β = 0.02 (*p* = 0.831), therefore men and women did not significantly differ on androgyny.

**FIGURE 2:**
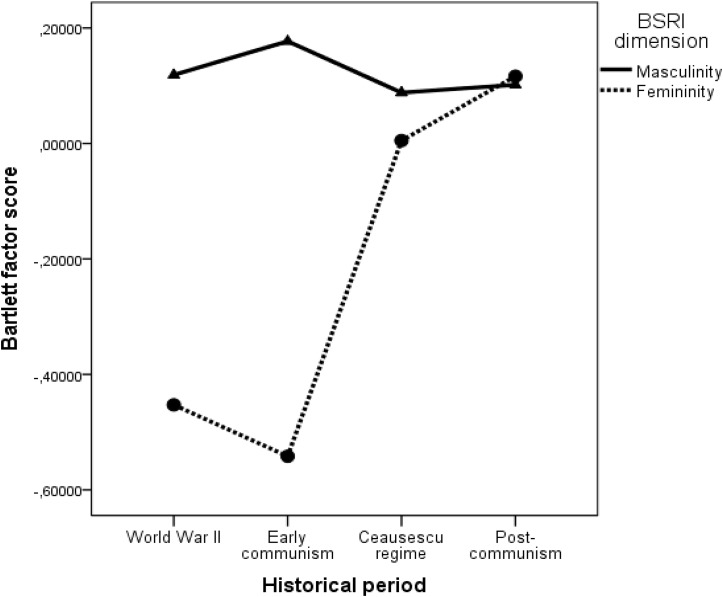
Evolution of masculinity and femininity in time.

Moreover, the historical period (year of death) had a positive and significant effect on femininity β = 0.24 (*p* = 0.009) but not on masculinity β = 0.06 (*p* = 0.465) showing that femininity increases in time, while masculinity did not show a significant increase in time. Therefore, we can conclude that Hypothesis 2 was also supported. The results are depicted in **Figure 2**.

As indicated in **Figure 2**, masculine attributes dominated during the period around the WWII and initial communist years, while the feminine attributes dominated in the late communist period. The results presented in **Table 3** showed that also androgyny had a significant positive association with the time passage β = 0.21 (*p* = 0.027). The positive and significant effect of historical period on androgyny is therefore likely to be explained by the increase in feminine attribute used in more recent years.

**FIGURE 3:**
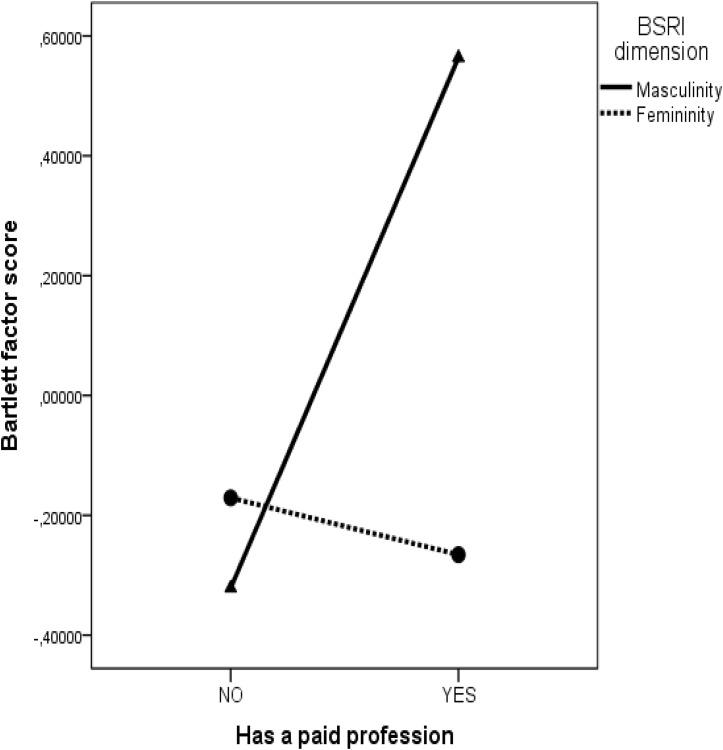
Masculinity and femininity as a function of paid profession.

**FIGURE 4:**
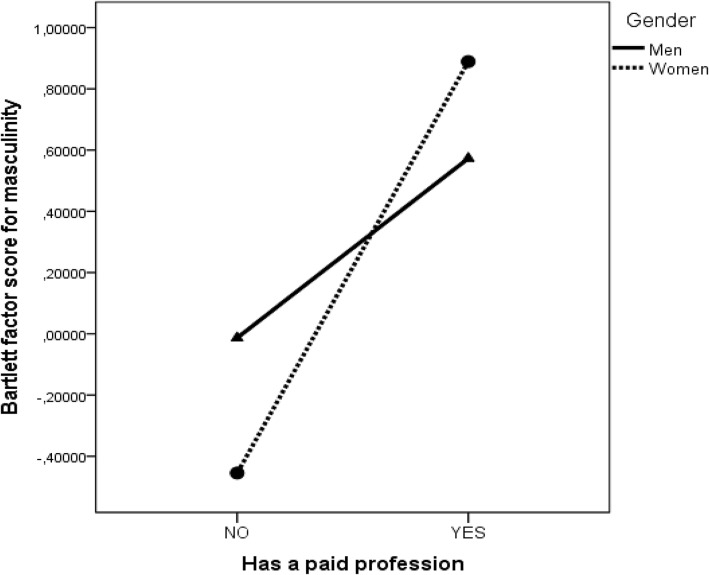
The interaction effect between gender and paid profession on masculinity.

Because the effect of profession on masculinity was positive and significant β = 0.20 (*p* = 0.011) and as depicted in **Figure 3**, people with a paid profession were described with masculine rather than feminine traits, we can conclude that Hypothesis 3 was also supported. Moreover, gender moderated the effect of paid profession on masculinity β = 0.27 (*p* = 0.001), in such a way that for women the difference between homemakers and the ones with a paid profession was larger than for men (the interaction effect is depicted in **Figure 4**).

**FIGURE 5:**
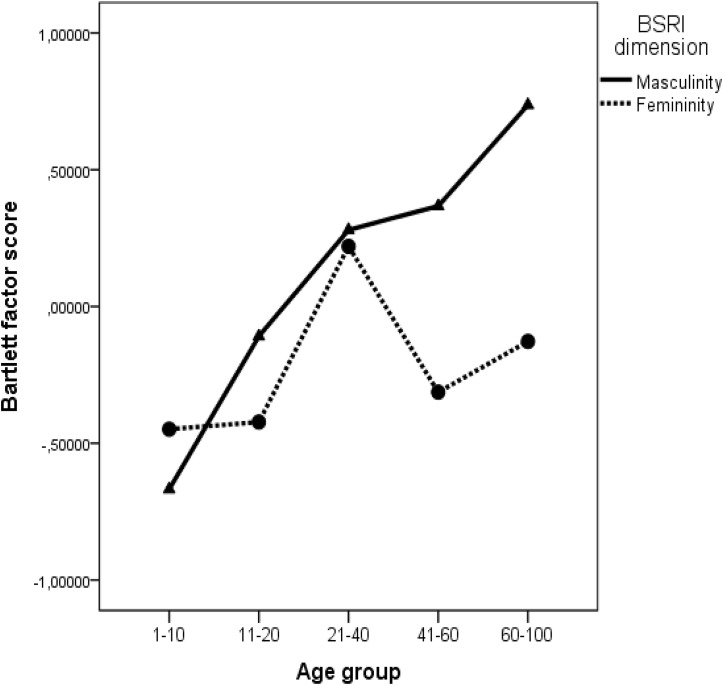
Evolution of masculinity and femininity with age.

Finally, the main effect of age on masculinity was significant β = 0.51 (*p* < 0.001) and as depicted in **Figure 5** masculinity scores increased with age, while femininity scores did not show a stable increase with age.

**FIGURE 6:**
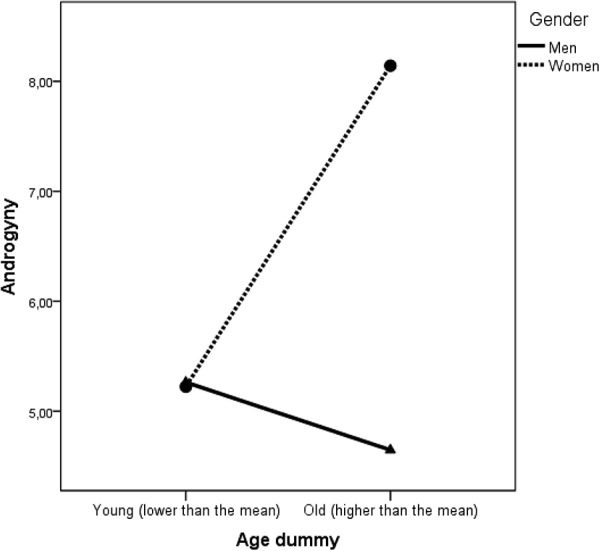
The interaction effect between age and gender on androgyny.

Moreover, our results show that the androgyny increased with age (β = 0.24; *p* = 0.001) as indicated in Model 1 presented in **Table 3**, therefore we can conclude that Hypothesis 4 was also supported. As illustrated in **Figure 6**, this effect was stronger for women than for men. We further analyzed the effect of gender for the two BSRI dimensions separately and as indicated in **Table 3**, age had a positive association with masculinity and not femininity.

Moreover, although age had a significant association with androgyny β = 0.24 (*p* = 0.001) (see Model 1 in **Table 3**), this effect was significantly moderated by gender β = 0.24 (*p* = 0.015) (see Model 2 in **Table 3**). After adding the cross-product term in Model 2, the main effect of age became not significant β = 0.07 (*p* = 0.498), while the interaction term between gender and age remained positive and significant. As depicted in **Figure 6**, androgyny increased with age for women, but not for men. Role accumulation seems therefore to go hand in hand with masculine traits as only these showed a significant positive association with age.

**FIGURE 7:**
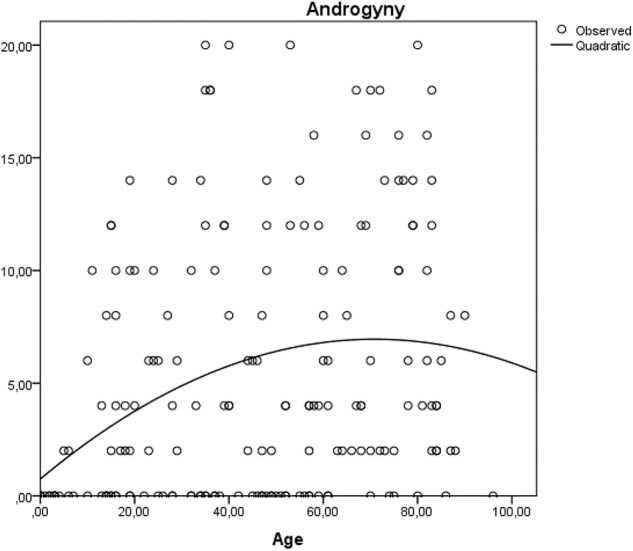
The non-linear association between age and androgyny.

Although the squared term of age was not significant, β = -0.12 (*p* = 0.070), the graphical depiction of the relationship between androgyny and age seems to reach indeed a plateau around the age of 60 as illustrated in **Figure 7**.

## Discussion

In our study, we have sampled the epitaphs of the Merry Cemetery of Săpânţa and analyzed the short biographies of 197 community members deceased between 1935 and 2010. Both masculine and feminine traits were coded for each of the sampled epitaphs. As most of the current research on the dynamic gender stereotypes stems from western urban contexts (Bosak et al., 2018) our study extends current research and calls for further exploration of dynamic gender stereotypes in a variety of social and historical contexts. Given the comprehensive nature of our data set we could explore the dynamic nature of gender stereotypes by simultaneously investigating three sources of change: (1) historical periods, (2) social roles effects, and (3) role accumulation across the lifespan.

### Historical Periods

First, we have investigated the influence of major historical events on the use of masculine and feminine traits and we have found that in situations of social unrest (WWII) community members were described using more masculine traits, while during the late communist period the feminine traits dominate. An important observation emerges from the analysis of the interaction effect depicted in **Figure 2**, namely that during the first years of communism masculine attributes seemed to be also dominant, fact that was probably associated with the resistance to the instauration of the new political regime. During the WWII and the first communist years, both men and women are predominantly described using masculine attributes. The social unrest that governed these historical periods probably pushed people to take more agentic and active (masculine) roles. On the one hand, men were actively involved in the conflicts in the public sphere while probably women had to take active roles and assume masculine attributes on the home front.

We argued that historical events change stereotypes via two mechanisms. On the one hand, critical events (wars, social conflicts) change the very nature of life in communities and as the actions and behaviors change, so do the gender-related attributes. On the other hand, critical events change the social structure and the institutional norms that are governing social behavior. With the change of social structure, comes a change in specific role prescriptions and role expectations. Our results show that for example during the late communist era, as the social equality propaganda and the myth of the heroic mother became widely spread after the 1970s (Curşeu and Boroş, 2011), the use of feminine attributes increased substantially and almost overruled the use of masculine ones that dominated during the previous times marked by war and social unrest (WWII and initial communist years). Our study is among the first to directly test the role of historical events on the dynamic nature of stereotypes and we call for more research that investigates the way in which critical events shape the content of gender stereotypes.

### Social Roles Effects

Second, we investigated the extent to which being employed impacts on the use of masculine traits and in line with previous research (Eagly and Steffen, 1984; March et al., 2016), we have found that employed people are described using more masculine traits than homemakers. This result is in line with the social role theory (Eagly, 2013) stating that the ascription of gender attributes is dependent on the social roles fulfilled by individuals. According to our results employed individuals are described by masculine rather than feminine traits, while the opposite is valid for homemakers. As illustrated by the positive effect of paid profession on androgyny, the role accumulation hypothesis is also supported by the fact that in general, employed members are described by more attributes than unemployed individuals.

Women had traditional professions like weaving and spinning and an illustrative example is presented in the following epitaph: “*Here I take my final rest/IH is my name/Already from my early childhood/I was very creative/My work made my exhibitions famous/Throughout the whole country/From Craiova to Bucuresti/I went to show my creations/Traditional rugs from Sãpân*ţ*a/I have made weaving and spinning an art/And I taught many girls to do it as well/I have made beautiful things/Which I sold throughout the country/And death found me in the capital/Where I came to sell my traditional rugs/Maybe I could have lived longer/But with sorrow I am telling you/My days were numbered/And my life faded away/I was taken from my dear ones/My husband is now alone/Two sons, daughters in law and grandchildren/Will all certainly miss me/I would also like to tell my colleagues/Preserve your traditional art/Don’t let it go to waste.*” Men on the other had had a wider range of professions from tractor drivers to engineers as illustrated by the following two epitaphs excerpts: “*Here I lie to take my rest/And SM is my name/Nobody should have the bad luck I had/I was a tractor driver/I death found me at my work/Far away from my village/I had to leave this life at a very early age/My poor mother/will never forget me/And she will grief me as long as she will live*” and “*As long as I lived/I have studied a lot/Graduated university with honors/Became a civil engineer/I had a good job at the municipality in Baia Mare/But the bad and ruthless death/ Put me to rest/And did not forgive me/Took my young life.*”

Only 50 epitaphs in our sample made explicit reference to paid professions, therefore a further clustering of the epitaphs using professional categories would have reduced the sample size for the professional categories. Although women had more traditionally feminine occupations, the ones that had a paid profession were described in more masculine terms than men having paid professions. We believe this result is in line with the role accumulation argument (Sieber, 1974) because in rural communities such as Săpânţa, the paid employment comes often on top of other social roles fulfilled in the household and community. Women with a paid profession are described as being masculine and given that older women are also more androgynous, these results indicate a significant role overload experienced by women in rural Romania. One epitaph excerpt clearly illustrates this dual role constraint: “*Here I lie to take my rest/And IS is my name/As long as I lived/I was blessed with golden hands/I sew blouses for girls/And I worked hard for many of them/So I could earn a living/And raise my five children/I raised them well/With God’s help/Two of my daughters married well/And also one of my sons/My kids were my joy in life/And I had to leave them too soon.*”

### Age Effects

We also show that as social roles accumulate during one’s lifetime so does the number of attributes used to describe the community member. Young infants were described by a rather small number of attributes and most certainly at young ages the feminine traits are dominant. A clear trend illustrated by our data is that masculine traits systematically increase with age. We believe this trend is aligned with the role accumulation hypothesis (Sieber, 1974; Strough et al., 2007), as individuals start taking active roles in the family and community they are described using more masculine traits. An interesting observation emerges from the additional analysis of the non-linear association between age and androgyny. As indicated in **Figure 7**, the role accumulation effect fades away after the age of 60 and it is conditional on gender. Androgyny seems to become more apparent for women as they grow older, but not for men. In other words, in this rural community, masculinity increases with age especially for women and this explains the interaction effect between gender and age on androgyny.

### Implications

We answer the call for more “ecological” investigations of how gender stereotypes really change in time (Diekman and Eagly, 2008; Haines et al., 2016; Ellemers, 2018) and our key contribution is that we explore a cultural artifact (a repository of social representations) and consider simultaneously various sources of stereotype change. By including gender, age, historical period and profession simultaneously as predictors, we could partial out their separate main influences. Moreover, as indicated by the regression analyses, masculinity is predicted by age and paid profession, while femininity by passage of time. The historical perspective taken here allows us to disentangle the role of ontogenesis and historical events in shaping the content of gender stereotypes. By including passage of time and the effect of age simultaneously in our analyses we disentangled the effect of ontogenesis-related factors versus social changes emerging during various time intervals.

Our study extends the research on the dynamic nature of stereotypes and opens valuable research venues, especially geared toward using secondary data or cultural artifacts for the exploration of how the content of gender stereotypes changes under the influence of various factors. First, we show that it is important to disentangle the historical period effects from the other factors that impact on the content change in stereotypes. These historical period effects however, capture the implications of various societal, historical and cultural characteristics. Therefore, our results point to the need of exploring various specific contextual variables like major social events and their direct effect on the content of gender stereotypes. The increased availability of secondary data in recent years through the internet and social media could, in principle, generate ample opportunities to analyze and capture the interplay between contextual changes the gender stereotypes in different cultures and societies.

Second, our study shows the relevance of evaluating time-bond biographical snapshots as indicators of gender stereotypes. Previous research on gender stereotypes in management used obituaries to explore differences in the way successful managers were described in western European countries (Kirchler, 1992; Rodler et al., 2001) as well as in communist and post-communist Romania (Curşeu and Boroş, 2011). As the content of the epitaphs and obituaries are likely to be influenced by social norms that also impact the stereotyping process in real social interactions (Radtke et al., 2000), these artifacts are accurate reservoirs of (gender) stereotypes. Similar approaches can be adapted to explore the way in which for example political regimes with their specific social structures and institutional profiles shape the content of gender or other social stereotypes.

### Limitations

Although we believe our study contributes to the research on dynamic stereotypes, it also has several limitations. First, the data used in this study is restricted to a particular community and it cannot be easily generalized. Second, the research is non-experimental and non-invasive, therefore no causal claims can be derived from our results. Third, although we have considered various factors that could impact on the use of feminine or masculine traits, the content of the epitaphs could have been shaped by other factors as well. We could not account for the process of writing the epitaphs. These are stylized summaries of one’s life and the artist who designed the cross and the family of the deceased member could in principle decide on the content of the epitaphs. One could argue that the epitaph contents are idealized depictions of one’s life, yet as clearly illustrated in the analyses reported in Curşeu and Pop-Curşeu (2011) an important cluster in the epitaph contents are the negative attributes (vices and other dysfunctional behaviors). Therefore, we cannot but assume that the epitaphs capture the quintessential attributes of the community members. The type of profession for example could be another relevant factor that could drive the content of gender stereotypes. Due to the rather small sample size for participants with a paid profession this analysis was not deemed appropriate in the context of our study.

## Conclusion

Our study uses a novel historic approach to investigate the dynamic nature of gender stereotypes in a small Romanian community. We have analyzed the gender stereotypes used (between 1935 and 2010) in the epitaphs written on the blue-painted crosses of the Merry Cemetery of Săpânţa. Our results support the effect of social roles on stereotypes showing that people with a paid profession tend to be described with masculine attributes. Moreover, our results support the role accumulation across the lifespan showing that androgyny increases with age, especially for women who are described as having more masculine attributes in late adulthood. While young children are mostly described using feminine attributes (both male and female) in late adulthood masculine attributes are dominant for both genders. Finally, our results open new venues for exploring the link between social change and the content of gender stereotypes by showing that during historical periods marked by social unrest masculine traits are dominant, while in non-conflictual historical periods feminine traits dominate, especially for women.

## Author Contributions

PLC and IP-C contributed to the design and writing of this study.

## Conflict of Interest Statement

The authors declare that the research was conducted in the absence of any commercial or financial relationships that could be construed as a potential conflict of interest.
